# Supplementary material for: Characterization of humoral and SARS-CoV-2 specific T cell responses in people living with HIV
Source: Nat Commun. 2021 Oct 5;12:5839. doi: 10.1038/s41467-021-26137-7 (PMC8492866; doi:10.1038/s41467-021-26137-7)
Supplement: Supplementary file 1 — Supplementary Information [file 41467_2021_26137_MOESM1_ESM.pdf]

Supplementary Information

Characterization of humoral and SARS-CoV-2 specific T cell responses in people living with HIV

Supplementary Fig.1: Antigen binding screen and associations between humoral responses and cohort parameters

Supplementary Fig.2: Magnitude of T cell responses and associations with HIV parameters, age, gender and ethnicity

Supplementary Fig.3: Interrelations between T cell and antibody responses in HIV positive and negative male donors

Supplementary Fig.4: Cytokine profile of SARS-CoV-2-, CMV- and Gag- specific T cells

Supplementary Fig.5: Association between T cell immunophenotyping and SARS-CoV-2 adaptive immune responses

Supplementary Fig.6: Frequencies of CXCR3 cTfh cells and associations with neutralization titers in HIV positive and negative donors

Supplementary Fig.7: Examples of gating strategies

Supplementary Table 1: Cohort demographics and clinical characteristics

Supplementary Table 2: Antibodies used for phenotypic analysis and virus-specific T cell characterization

Supplementary Fig.1: Antigen binding screen and associations between humoral responses and cohort parameters. a Antigen binding screen in pre-pandemic samples from n=16 HIV positive donors and **b** the whole cohort with convalescent COVID-19 disease (n=82). Dotted lines indicate negative, low positive and positive threshold for absorbance [450nm]. **c** Correlation between age and S1 IgG titer according to gender in HIV negative donors and HIV positive subjects. **d** Correlation between age and ID50 according to gender in HIV negative donors and HIV positive subjects and **e** between DPSO and ID50 in the two study groups. **f** S1 IgG titer and ID50 levels summary dot plots according to ethnicity in HIV positive (n=30) and negative donors (n=29). Line on the plot represents the Geometric Mean. **g** S1 IgG, N titers and ID50 according to gender in HIV positive (n=29) and HIV (n=29) negative donors. S1 IgG HIV-Female vs HIV+ Female p=0.0110; HIV- Male vs HIV+ Female p=0.02; HIV+ Male vs HIV+ Female p=0.004). Plots show the Geometric Mean. Significance determined by two-tailed Mann-Whitney test. The non-parametric Spearman test was used for correlation analysis. *p < 0.05, **p<0.01.

Supplementary Figure 1

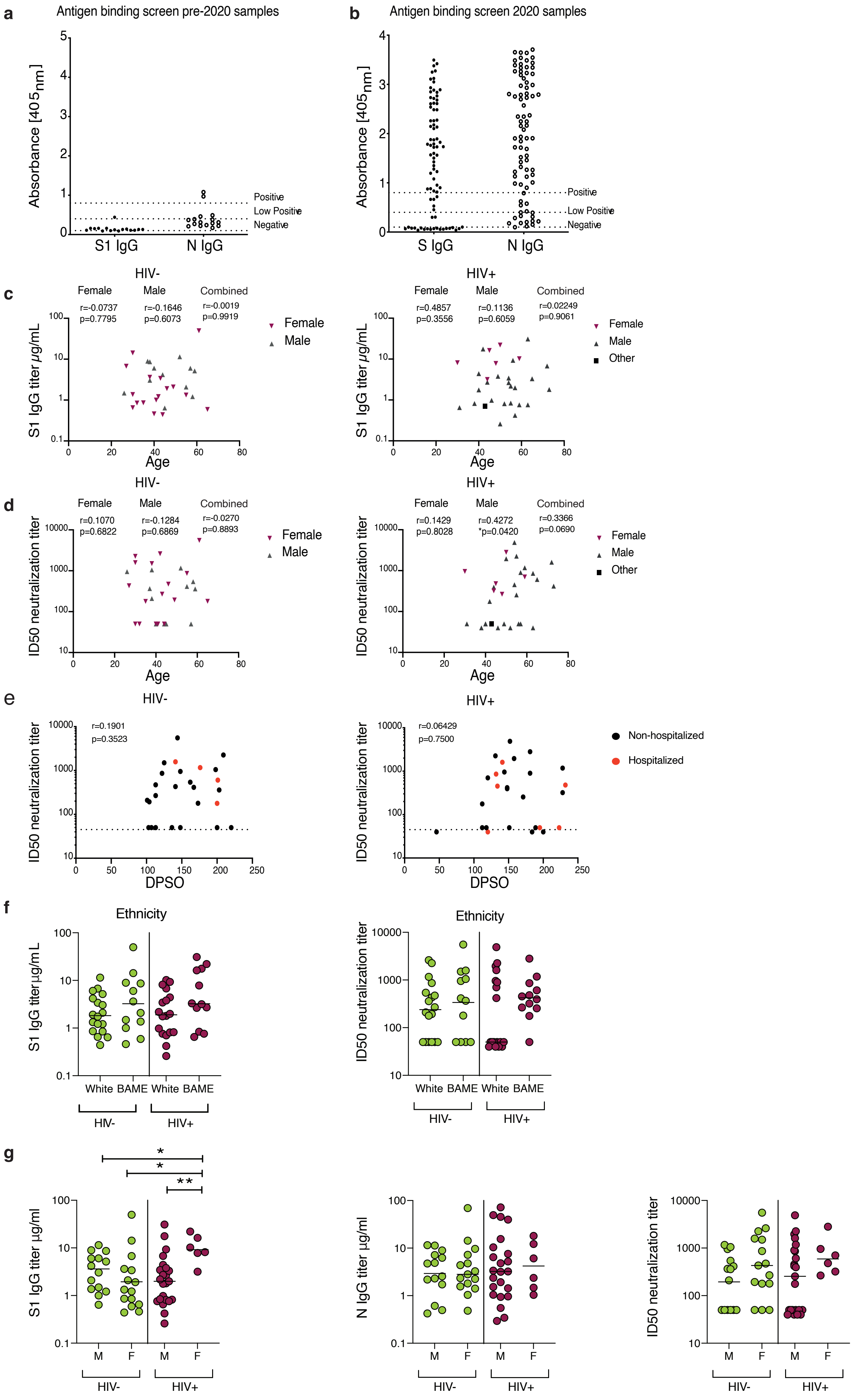

Supplementary Fig.2: Magnitude of T cell responses and associations with HIV parameters, age, gender and ethnicity. a Percentage of responders to each peptide pool **b** Magnitude of the INF-γ-ELISpot responses. IFN-γ SFU/10^6 PBMCs are shown for SARS-CoV-2 ORF3a, ORF6, ORF7, ORF8 and Env between HIV negative (green) and HIV positive (red). (n=30 per group). Plots show the Geometric Mean**. c** Magnitude of the total SARS-CoV-2 responses analyzed in pre-pandemic samples, confirmed SARS-CoV-2 and suspected cases with clinical definition but found to be SARS-CoV-2 seronegative on screening. In suspected cases, orange dots depict HIV negative and brown dots HIV positive donors. Error bars represent Mean ±SD (pre-pandemic vs HIV-COVID p=0.0192, pre-pandemic vs HIV+COVID p= 0.0278, COVID suspected vs HIV-COVID p= 0.0021, COVID suspected vs HIV+COVID p= 0.0036). Correlation between CD4:CD8 ratio in HIV infected individuals with their **d** Nucleoprotein and **e** Membrane responses, depicting disease severity per donor. Correlation of total SARS-CoV-2 responses with age in **f** HIV negative (n=30) and **g** HIV positive (n=30), depicting disease severity per donor. **h** Correlation of total SARS-CoV-2 responses with DPSO in HIV negative and **i** HIV positive, depicting disease severity per donor. **j** Magnitude of the total SARS-CoV-2 responses by ethnicity (n=30 in each group) and **k** gender between HIV negative (n=30 in total) and HIV positive (n=29 in total) donors. **l** Spike, M and N-specific T cell responses and **m** diversity of the IFN-γ-ELISpot responses between HIV negative (n=30) and positive donors (n=30) according to gender. Geometric Mean is shown on the plots. The non-parametric Spearman test was used for correlation analysis (two-tailed). Two-way ANOVA was used for group comparisons. *p < 0.05, **p<0.01.

Supplementary Figure 2

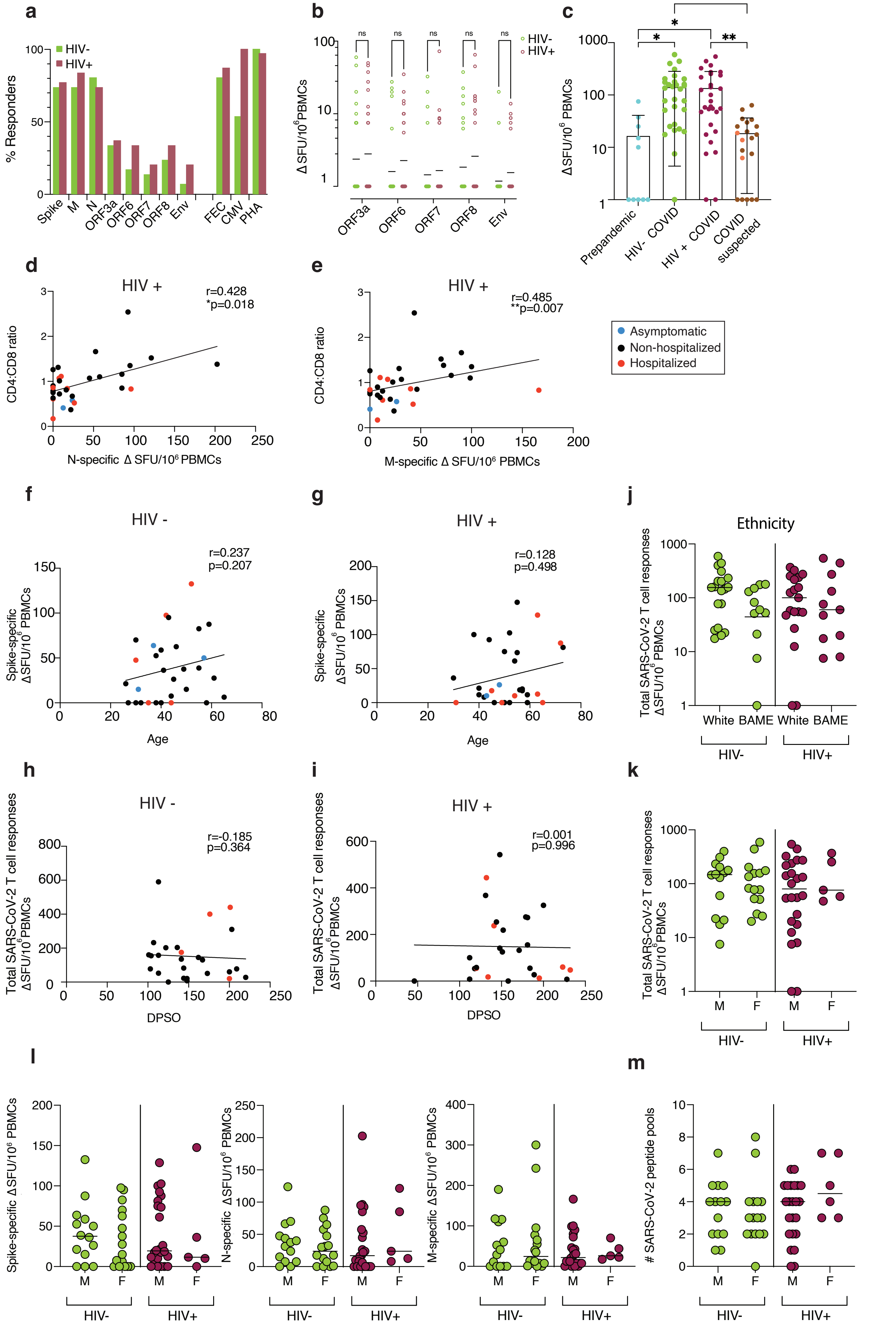

Supplementary Fig.3: Interrelations between T cell and antibody responses in HIV positive and negative male donors. **a** Correlation of total SARS-CoV-2 responses with S1 IgG titers in HIV negative and **b** HIV positive males. **c** Correlation of total SARS-CoV-2 responses with N IgG titers in HIV negative and **d** HIV positive males. **e** Correlation of neutralization titers with Spike-specific SARS-CoV-2 responses in HIV negative and **f** HIV positive males. **g** Correlation of neutralization titers with total SARS-CoV-2 responses in HIV negative and **h** HIV positive males. Red dots: hospitalized cases; black dots: non-hospitalized cases. The non-parametric Spearman test was used for correlation analysis. Two-tailed $*p < 0.05$.

Supplementary Figure 3

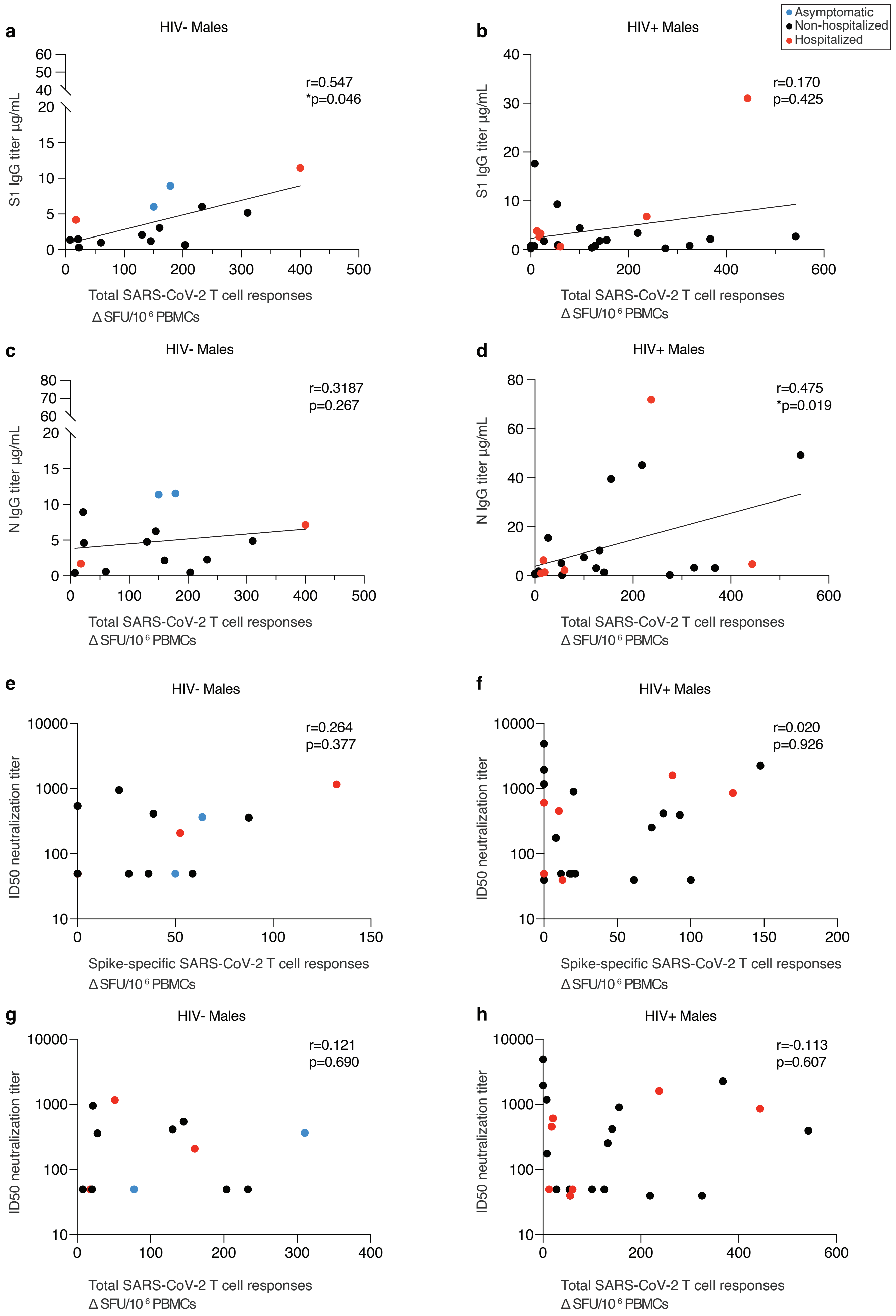

Supplementary Fig.4: Cytokine profile of SARS-CoV-2-, CMV- and Gag- specific T cells. **a** Frequency of SARS-CoV-2-specific CD154$^+$ CD4 T cells identified by expression of IFN-γ$^+$, IL-2$^+$, or TNF-α$^+$ or overall responses with at least one of the three cytokines (IFN-γ, TNF-α and IL-2) against Spike (S1 and S2 pools) **b** M/N or **c** combined (Spike and M/N) responses in HIV-negative (HIV-, n=12) and HIV positive individuals (HIV+, n=11) recovering from COVID-19 disease. **d** Representative flow plots and summary data **e** showing frequencies of overall (CD154$^+$IFN-γ$^+$, CD154$^+$IL-2$^+$, or CD154$^+$TNF-α$^+$) SARS-CoV-2-, CMV-, or Gag-specific CD4 T cell responses in n=12 HIV- and n=11 HIV+ donors (HIV- SARS-CoV-2 vs CMV p=0.0468, HIV+ SARS-CoV-2 vs CMV p=0.0137, HIV+ SARS-CoV-2 vs Gag p=0.001, HIV+ CMV vs Gag p=0.0225). **f** Frequency of SARS-CoV-2-specific CD8 T cells identified by expression of IFN-γ$^+$, IL-2$^+$ or TNF-α$^+$, or overall responses with at least one of the three cytokines (IFN-γ, TNF- α and IL-2) against Spike (S1 and S2 pools) **g** M/N or **h** combined (Spike and M/N) responses in HIV-negative (HIV-, n=12) and HIV-positive individuals (HIV+, n=11). (Non-spike-specific IL-2+ CD8 T cels, p= 0.0295; Total SARS-CoV-2 specific IL-2+ CD8 T cells, p= 0.0335). **i** Representative flow plots and **j** summary data showing frequencies of overall (IFN-γ$^+$, IL-2$^+$, or TNF-α$^+$) SARS-CoV-2-, CMV-, or Gag-specific CD8 T cell responses in the HIV- (n=12) and HIV+ (n=11) group. (HIV- SARS-CoV-2 vs CMV p=0.0312, HIV+ SARS-CoV-2 vs CMV p=0.001, HIV+ SARS-CoV-2 vs Gag p=0.001, HIV+ CMV vs Gag p=0.009). Error bars represent SEM. The non-parametric Spearman test was used for correlation analysis; p values for individual correlation analysis within groups, HIV- (green) or HIV+ (red) or combined correlation analysis (black) are presented. Significance determined by Mann-Whitney *U* test or Wilcoxon matched- pairs signed rank test, Two-tailed *p<0.05, **p<0.01, ***p < 0.001.

Supplementary Figure 4

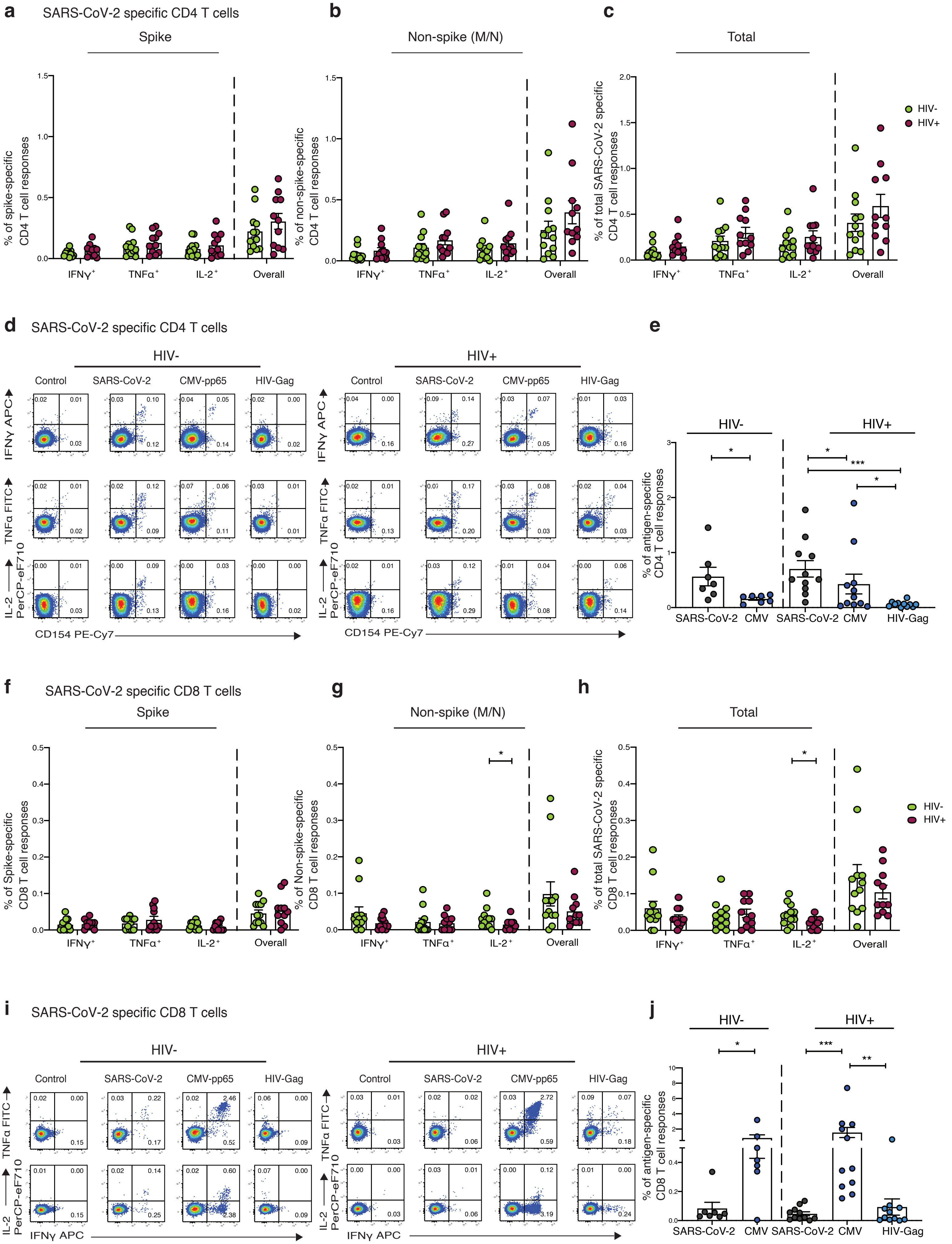

Supplementary Fig.5: Association between T cell immunophenotyping and SARS-CoV-2 adaptive immune responses. **a** Representative flow plots and **b** summary data of the frequency of CD38$^+$ HLADR$^+$ CD4 (p=0.0034) and CD8 (p=0.0252) T cells, and correlations between percentage of CD38$^+$ HLADR$^+$ CD4 and CD8 T cells and total SARS-CoV-2-specific T cell responses in HIV-seronegative (HIV-, n=26) and HIV positive individuals (HIV+, n=19). Plots show the Geometric Mean. **c** Representative flow plots and **d** summary data of frequency of PD-1$^+$ TIGIT$^+$ CD4 (p=0.0003) and CD8 (p=0.0238) T cells, and correlations between percentage of PD-1$^+$ TIGIT$^+$ CD4 and CD8 T cells and total SARS-CoV-2-specific T cell responses. Plots show the Geometric Mean. **e** Representative flow plots showing the gating strategy used to define total circulating and activated Tfh subsets in the study groups and summary data (CXCR5$^+$PD-1$^+$Tfh p=0.0025). Plots show the Geometric Mean. **f** Correlations between percentage of activated Tfh and S1 IgG or N IgG titers. Significance determined by two-tailed Mann-Whitney *U* test. The non-parametric Spearman test was used for correlation analysis; combined correlation analysis is depicted. *p<0.05, **p<0.01, ***p < 0.001.

Supplementary Figure 5

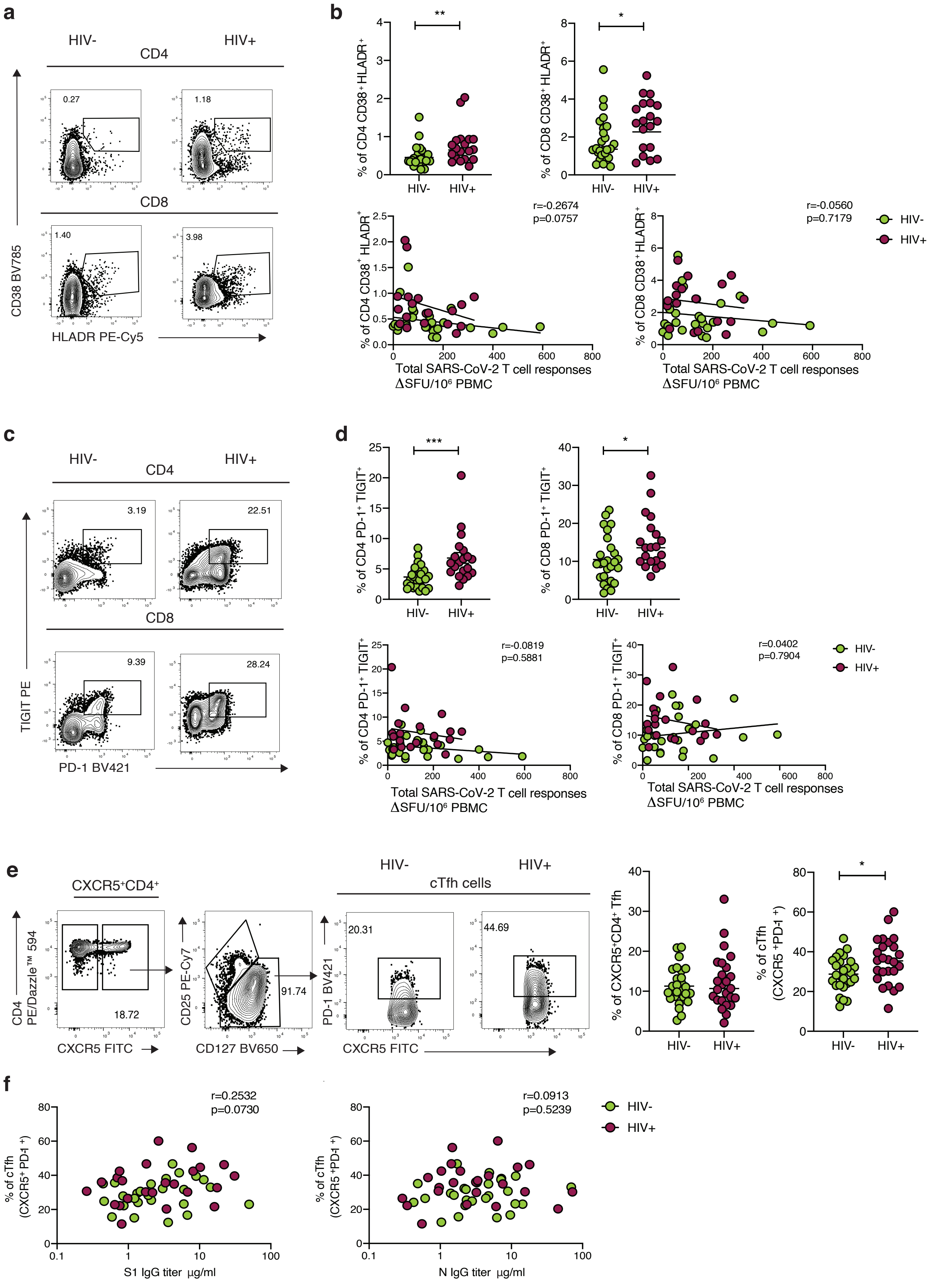

Supplementary Fig.6: Frequencies of CXCR3 cTfh cells and associations with neutralization titers in HIV positive and negative donors. a Representative flow plots of the gating strategy for the identification of CXCR3^{+} and CXCR3^{-} cTfh cells (PD-1^{+}CD25^{low}CD127^{high}CXCR5^{+}CD4^{+}). **b** Summary data of the proportions of CXCR3^{+} and CXCR3^{-} out of total cTfh cells in SARS-CoV-2 in convalescent HIV negative (n=27) and HIV positive (n=25) individuals. (CXCR3+ p=0.0201, CXCR3- p=0.0182) **c** frequency of CXCR3^{+} and CXCR3^{-} cTfh cells between HIV negative (n=27) and positive (n=24) donors according to gender. (CXCR3^{+}: HIV- Male vs Female p=0.0321, HIV- Female vs HIV+ Male p=0.0027; CXCR3^{-}: HIV- Male vs Female p=0.0321, HIV- Female vs HIV+ Male p=0.0021) **d** Correlation between ID50 and frequency of CXCR3^{+} cTfh cells and **e** CXCR3^{-} cTfh cells. **f** Correlation between ID50 and percentage of CXCR3^{+} cTfh cells according to gender in HIV negative (n=27) and **g** HIV positive (n=23) donors. **h** Correlation between ID50 and percentage of CXCR3^{-} cTfh cells according to gender in HIV negative (n=27) and **i** HIV positive (n=23) donors. Significance determined by two-tailed Mann-Whitney *U* test. The non-parametric Spearman test was used for correlation analysis. *p<0.05, **p<0.01.

Supplementary Figure 6

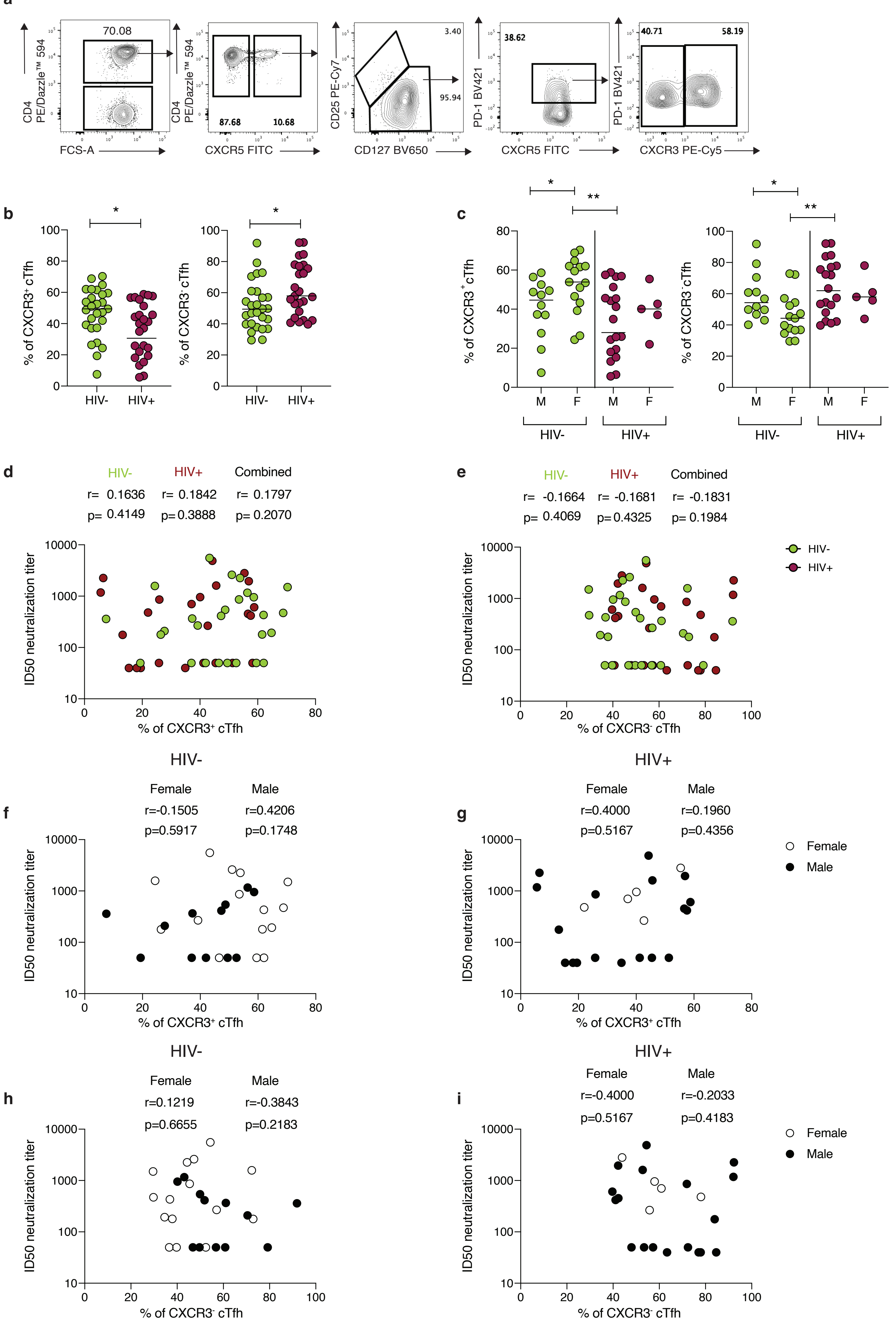

Supplementary Fig.7: Examples of gating strategies. **a** Cells were gated on single lymphocyte population by side and forward side scatter, followed by live cells gating by excluding dead, CD19 and CD14 cells. Following gating on live CD3+ T cells, cells were gated on CD4 T cells (CD4+CD8-) or CD8 T cells (CD4-CD8+). Virus-specific CD4 and CD8 T cells were identified by expression of CD154, IFN-γ, TNF and IL-2 as shown following peptide stimulation. This gating strategy was used for Fig. 4a, d, 5a, c, e and Supplementary Fig. 4d, i. **b** Gating strategy used to identify the proportion of CD45RA-/CCR7+ central memory (CM), CD45RA+/CCR7+ naïve, CD45RA+/CCR7- terminally differentiated effector memory (TEMRA) and CD45RA-/CCR7- effector memory (EM) CD4 and CD8 T cell subsets. This gating strategy was used for Fig. 6b, c. **c** Gating strategy used for T cell immunophenotyping. This gating strategy was used for Supplementary Fig. 5a, c. **d** Gating strategy used for the identification of cTfh cells (PD-1^{+}CD25^{low}CD127^{high}CXCR5^{+}CD4^{+}). This gating strategy was used for Supplementary Fig. 5e and Fig. 6a.

Supplementary Figure 7

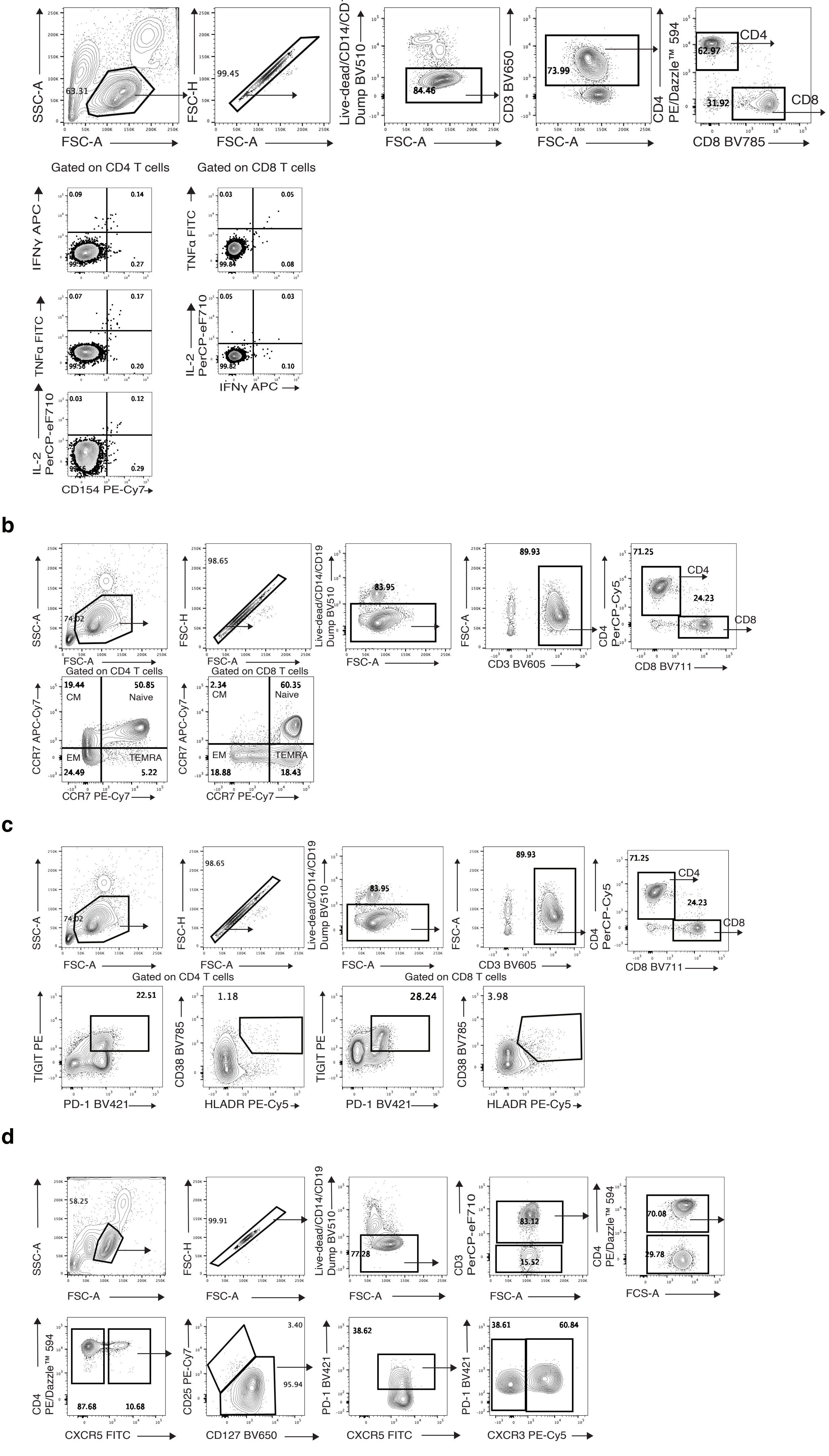

Supplementary Table 1. Cohort Demographics and Clinical Characteristics

		**HIV+, Lab confirmed SARS-CoV-2** RT-PCR+ and/or Ab Pos	**HIV+ *Suspected SARS-CoV-2**	**HIV- Lab confirmed SARS-CoV-2** RT-PCR+ and/or Ab Pos	**HIV- *Suspected/ Household**	**HIV+ pre-pandemic**
**Group size**	n	24	23	31	4	16
	SARS-CoV-2 seropositive at date of sampling, n (%)	23 (95.8)	7 (30.43)	29 (93.5)	0 (0.0)	0 (0.0)
**COVID-19 severity****	Asymptomatic (score 1), n (%)	2 (8.3)	0 (0.0)	4 (12.9)	1 (25.0)	-
	Non-hospitalized (score 2), n (%)	14 (58.3)	22 (95.7)	23 (74.2)	3 (75.0)	-
	Hospitalized (score 4-5), n (%)	8 (33.3)	1 (4.3)	4 (12.9)	0 (0.0)	-
**COVID-19 symptoms**	Fever, n (%)	13 (54.2)	18 (78.3)	16 (51.6)	2 (50.0)	-
	Shortness of breath, n (%)	14 (58.3)	14 (60.9)	12 (38.7)	1 (25.0)	-
	Fatigue, n (%)	21 (87.5)	20 (87.0)	20 (64.5)	4 (100.0)	-
	Cough, n (%)	17 (70.8)	18 (78.3)	12 (38.7)	3 (75.0)	-
	Headache, n (%)	10 (41.7)	14 (60.9)	13 (41.9)	2 (50.0)	-
	Altered taste/smell, n (%)	13 (54.2)	10 (43.5)	22 (71.0)	2 (50.0)	-
	Duration of symptoms in days, median (range)	14 (2-48)	21 (3-84)	10 (3-30)	7 (5-7)	-
	Days post-symptom onset (DPSO), median (range)	148 (46-232)	181 (126-273)	144 (101-220)	200 (125-203)	-
**Risk factors**	Age, median (range)	51 (30-73)	53 (30-67)	42 (26-65)	35.5 (30-41)	48 (30-60)
	Sex, n female:male:other	4:19:1	2:21:0	16:15:0	2:2:0	2:14:0
	BMI, median (range)	27.3 (20.2-33.8)	24.6 (20.3-39.4)	25.7 (19.1-37.7)	21.9 (20.0-27.6)	
**Ethnicity**	White, n (%)	16 (66.7)	17 (73.9)	19 (61.3)	1 (25.0)	11 (68.8)
	BAME, n (%)	8 (33.3)	6 (26.1)	12 (38.7)	3 (75.0)	5 (31.3)
**Smoking**	Current, n (%)	3 (12.5)	4 (17.4)	5 (16.1)	0 (0.0)	2 (12.5)
	Ex-smoker, n (%)	1 (4.2)	1 (4.3)	2 (6.5)	0 (0.0)	1 (6.3)
	Non-smoker, n (%)	20 (83.3)	18 (78.3)	24 (77.4)	4 (100.0)	13 (81.3)
**HIV parameters**	HIV viral load	<50	<50	-	-	<50
	CD4, median (range)	571 (133-1110)	586 (310-1360)	-	-	590 (350-940)
	CD4:CD8, median (range)	0.84 (0.17-2.54)	0.97 (0.37-2.15)	-	-	0.82 (0.43-2.26)
**Pre-existing conditions**	None, n (%)	10 (41.7)	8 (34.8)	15 (48.4)	4 (100.0)	11 (68.8)
	Diabetes, n (%)	6 (25.0)	1 (4.3)	1 (3.2)	0 (0.0)	1 (6.3)
	Hypertension/CVD, n (%)	7 (29.2)	2 (8.7)	3 (9.7)	0 (0.0)	2 (12.5)
	Renal disease, n (%)	2 (8.3)	2 (8.7)	0 (0.0)	0 (0.0)	0 (0)
	Respiratory disease (asthma and COPD), n (%)	2 (8.3)	4 (17.4)	1 (3.2)	0 (0.0)	0 (0)
	Liver disease, n (%)	1 (4.2)	2 (8.7)	0 (0.0)	0 (0.0)	1 (6.3)
	Other	ITP, psoriasis, pituitary gland failure	Osteoporosis, peripheral neuropathy, hypothyroidism	RA, iron deficiency, psoriasis, gout, SLE, hypothyroidism		

*Confirmed, possible and probable cases specified by the case definition for COVID-19, as of 29 May 2020, European Centre for Disease Prevention and Control [https://www.ecdc.europa.eu/en/covid-19/surveillance/case-definition]

**Severity of COVID-19 was classified according to the WHO (World Health Organisation) clinical progression scale (Aitken et al ., 2020).

Supplementary Table 2

Fluorochrome-Conjugated Antibodies:

Antibodies	Supplier	Identifier	Clone	Dilution
APC/Cyanine7 anti-human CD19 Antibody	BioLegend	Cat # 363010	Clone # SJ25C1	1 in 100ul
APC/Cy7 anti-human CD197 (CCR7)	BioLegend	Cat # 353212	Clone # G043H7	1 in 50ul
Brilliant Violet 605™ anti-human CD3 Antibody	BioLegend	Cat # 317322	Clone # OKT3	1 in 100ul
Brilliant Violet 650™ anti-human CD127 (IL-7Rα) Antibody	BioLegend	Cat # 351325	Clone # A019D5	1 in 100ul
Brilliant Violet 650™ anti-human CD3 Antibody	BioLegend	Cat # 317324	Clone # OKT3	1 in 100ul
Brilliant Violet 711™ anti-human CD27 Antibody	BioLegend	Cat # 302833	Clone # O323	1 in 100ul
Brilliant Violet 785™ anti-human CD38 Antibody	BioLegend	Cat # 303530	Clone # HIT2	1 in 50ul
Alexa Fluor® 700 anti-human CD45RA Antibody	BioLegend	Cat # 304120	Clone # HI100	1 in 50ul
PE/Cyanine7 anti-human CD45RA Antibody	BioLegend	Cat # 304126	Clone # HI100	1 in 200ul
Brilliant Violet 421™ anti-human CD279 (PD-1) Antibody	BioLegend	Cat # 329920	Clone # EH12.2H7	1 in 100ul
PE/Dazzle™ 594 anti-human CD4 Antibody	BioLegend	Cat # 300548	Clone # RPA-T4	1 in 100ul
PE anti-human IgD Antibody	BioLegend	Cat # 348204	Clone # IA6-2	1 in 200ul
APC anti-human IFN-γ Antibody	BioLegend	Cat # 506510	Clone # B27	1 in 100ul
Brilliant Violet 785™ anti-human CD8a Antibody	BioLegend	Cat # 301046	Clone # RPA-T8	1 in 100ul
Brilliant Violet 711™ anti-human CD8a Antibody	BioLegend	Cat # 301044	Clone # RPA-T8	1 in 100ul
Brilliant Violet 510™ anti-human CD4 Antibody	BioLegend	Cat # 300546	Clone # RPA-T4	1 in 200ul
Brilliant Violet 510™ anti-human CD14 Antibody	BioLegend	Cat # 301842	Clone # M5E2	1 in 200ul

Brilliant Violet 510™ anti-human CD19 Antibody	BioLegend	Cat # 302242	Clone # HIB19	1 in 200ul
Brilliant Violet 711™ anti-human CD279 (PD-1) Antibody	BioLegend	Cat # 329928	Clone # EH12.2H7	1 in 50ul
PE/Cyanine7 anti-human CD154 Antibody	BioLegend	Cat # 310832	Clone # 24-31	1 in 200ul
APC-R700 Mouse Anti-Human CD196 (CCR6)	BD Biosciences	Cat # 565173	Clone # 11A9	1 in 25ul
BB515 Rat Anti-Human CXCR5 (CD185)	BD Biosciences	Cat # 564624	Clone # RF8B2	1 in 50ul
BV605 Mouse Anti-Human CD56	BD Biosciences	Cat # 562780	Clone # NCAM16.2	1 in 50ul
PE-Cy7 Mouse Anti-Human CD25	BD Biosciences	Cat # 335824	Clone # 2A3	1 in 50ul
BB700 Mouse Anti-Human CD4	BD Biosciences	Cat # 566393	Clone # SK3	1 in 200ul
PE-Cy™5 Mouse Anti-Human CD183	BD Biosciences	Cat # 551128	Clone # 1C6/CXCR3	1 in 25ul
PE-Cy™5 Mouse Anti-Human HLA-DR	BD Biosciences	Cat # 562007	Clone # G46-6	1 in 200ul
FITC Mouse Anti-Human TNF-α	BD Biosciences	Cat # 554512	Clone # MAb11	1 in 400ul
BV421 Mouse Anti-Human CD107A	BD Biosciences	Cat # 562623	Clone # H4A3	1 in 200ul
Alexa Fluor® 700 Mouse anti-Human Granzyme B	BD Biosciences	Cat # 560213	Clone # GB11	1 in 100ul
PerCP-eFluor 710 Anti-Human IL-2	eBioscience	Cat # 46-7029-42	Clone # MQ1-17H12	1 in 50ul
PerCP-eFluor 710 Anti-Human CD3	eBioscience	Cat # 46-0037-42	Clone # OKT3	1 in 100ul
PE Anti-Human TIGIT	eBioscience	Cat # 12-9500-42	Clone # MBSA43	1 in 100ul

Key Chemicals, Peptides, and Commercial Assays

Reagents	Supplier	Identifier
PepTivator SARS-CoV-2 Prot_N	Miltenyi Biotec	Cat # 130-126-698
PepTivator SARS-CoV-2 Prot_M	Miltenyi Biotec	Cat # 130-126-702
PepTivator CMV pp65, human	Miltenyi Biotec	Cat # 130-093-438
ProMixTM HIV Peptide Pool	Proimmune	Cat # PX-HIV
Human IFN-γ ELISpot Kit	Mabtech	Cat # P3420-2A

Human Anti-Cytomegalovirus IgG ELISA Kit (CMV)	Abcam	Cat # ab108724
Brefeldin A	eBioscience	Cat # 00-4506-51
GolgiStop (with Monensin)	BD Biosciences	Cat # 554724
Foxp3/TF Staining Buffer Set	Invitrogen	Cat # 00-5523-00
BD Cytofix/Cytoperm™ Fixation/Permeabilization Solution Kit	BD Biosciences	Cat # 554714
