# Supplementary material for: Characterization of humoral and SARS-CoV-2 specific T cell responses in people living with HIV
Source: Nat Commun. 2021 Oct 5;12:5839. doi: 10.1038/s41467-021-26137-7 (PMC8492866; doi:10.1038/s41467-021-26137-7)
Supplement: Supplementary file 3 — Reporting summary [file 41467_2021_26137_MOESM3_ESM.pdf]

nature research

Corresponding author(s): Laura E. McCoy and Dimitra Peppa

Last updated by author(s): Sep 1, 2021

Reporting Summary

Nature Research wishes to improve the reproducibility of the work that we publish. This form provides structure for consistency and transparency in reporting. For further information on Nature Research policies, see our Editorial Policies and the Editorial Policy Checklist.

Statistics

For all statistical analyses, confirm that the following items are present in the figure legend, table legend, main text, or Methods section.

n/a	Confirmed	
☐	☒	The exact sample size (*n*) for each experimental group/condition, given as a discrete number and unit of measurement
☐	☒	A statement on whether measurements were taken from distinct samples or whether the same sample was measured repeatedly
☐	☒	The statistical test(s) used AND whether they are one- or two-sided<br>*Only common tests should be described solely by name; describe more complex techniques in the Methods section.*
☐	☒	A description of all covariates tested
☐	☒	A description of any assumptions or corrections, such as tests of normality and adjustment for multiple comparisons
☐	☒	A full description of the statistical parameters including central tendency (e.g. means) or other basic estimates (e.g. regression coefficient) AND variation (e.g. standard deviation) or associated estimates of uncertainty (e.g. confidence intervals)
☐	☒	For null hypothesis testing, the test statistic (e.g. *F*, *t*, *r*) with confidence intervals, effect sizes, degrees of freedom and *P* value noted<br>*Give P values as exact values whenever suitable.*
☒	☐	For Bayesian analysis, information on the choice of priors and Markov chain Monte Carlo settings
☒	☐	For hierarchical and complex designs, identification of the appropriate level for tests and full reporting of outcomes
☐	☒	Estimates of effect sizes (e.g. Cohen's *d*, Pearson's *r*), indicating how they were calculated

Our web collection on statistics for biologists contains articles on many of the points above.

Software and code

Policy information about availability of computer code

Data collection	S1 and N IgG titers measurement: MultiskanFC (Thermofisher) plate reader and S1 & N-specific IgG titers interpolated from the IgG standard curve using 4PL regression curve-fitting on GraphPad Prism 8.<br><br>Antibody neutralization assay: Glomax luminometer (Promega).<br><br>Flow cytometric assays: BD Fortessa X20 using BD FACSDiva8.0 (BD Bioscience).<br><br>IFN-γ ELISpot Assay: automated ELISpot Reader System (Autoimmun Diagnostika GmbH), AID ELISPot 7.0
Data analysis	Flow cytometry data were analyzed with FlowJo10(Treestar)<br>Prism 8 (GraphPad Software)<br>SPICE version 6.0.<br>Cytobank platform (https://cytobank.org)

For manuscripts utilizing custom algorithms or software that are central to the research but not yet described in published literature, software must be made available to editors and reviewers. We strongly encourage code deposition in a community repository (e.g. GitHub). See the Nature Research guidelines for submitting code & software for further information.

Data

Policy information about availability of data

All manuscripts must include a data availability statement. This statement should provide the following information, where applicable:

- Accession codes, unique identifiers, or web links for publicly available datasets
- A list of figures that have associated raw data
- A description of any restrictions on data availability

The source data from all the main and supplementary figures are included in the Source Data file and will be made available with publication.

Field-specific reporting

Please select the one below that is the best fit for your research. If you are not sure, read the appropriate sections before making your selection.

- [x] Life sciences
- [ ] Behavioural & social sciences
- [ ] Ecological, evolutionary & environmental sciences

For a reference copy of the document with all sections, see nature.com/documents/nr-reporting-summary-flat.pdf

Life sciences study design

All studies must disclose on these points even when the disclosure is negative.

Sample size

A total of n=47 HIV positive and n=35 HIV negative subjects with recovered confirmed and/or suspected COVID-19 disease were included. Sixteen demographically age-, sex- and lifestyle-matched HIV-1 seropositive individuals were included for comparison, from whom samples were collected between (February 2017-November 2019; pre-pandemic). Sample sizes were based on available sample sets with available clinical details.

Data exclusions

All subjects recruited in the study were included. No exclusion criteria were established prior to testing. HIV-negative and HIV-positive participants with recovered confirmed and/or suspected COVID-19 disease were screened for SARS-CoV-2 seropositivity. SARS-CoV-2 IgG seropositivity was determined using standardized ELISA assay as previously described in published methods (https://journals.plos.org/plospathogens/article id=10.1371/journal.ppat.1008817; https://science.sciencemag.org/content/370/6522/1339). Individuals with suspected SARS-CoV-2 infection who were found to be seronegative (i.e undetectable SARS-CoV-2 antibodies to S1 and N antigens, Figure 1a and Supplementary Figure 1a-b and 2c) were not included in subsequent analyses of confirmed SARS-CoV-2 cases (Figure 1b-g, Figure 2-6, Supplementary Figure 1c-g, Supplementary Figure 2a,b,c-m, Supplementary Figure 3-6). A total of n=4 of HIV-negative individuals and n=16 of HIV-positive individuals were found to be SARS-CoV-2 seronegative (Figure 1a and Supplementary Figure 1a-b and 2c).

For IFN-γ-ELISpot assays, if the responses were found to be lower than two standard deviations of the sample specific control or lower than 5 SFU/106 PBMCs, results were excluded. Also, if the responses to positive control wells (PHA, FEC) were negative, the results were excluded from further analysis.

Intracellular cytokine stimulation (ICS) functional assay was performed in a sub-group of HIV positive (n=11) and HIV negative (n=12) donors with available PBMC and detectable responses by IFN-γ-ELISpot.

For male/female comparison: one HIV-positive individual who was classified under “other” was not included in the analysis.

Replication

The samples for standardized semiquantitative ELISA for S1 and N were run in technical duplicate and all attempts were successful. Pre-2020 samples were used to establish a threshold for the seropositivity screening assays as previously described in published methods (https://journals.plos.org/plospathogens/article id=10.1371/journal.ppat.1008817 and https://science.sciencemag.org/content/370/6522/1339)

Standardized ELISpot assays were run in technical duplicates for all donors with available PBMCs and all attempts were successful. Each ELISpot plate/batch contained an internal pre-characterized QC sample (internal control) with defined acceptance range for T cell responses to control peptide pools in addition to appropriate negative and positive controls included for each donor tested. Due to limited sample availability ELISpot assay replication for individual study donors was not feasible.

Results from phenotypic analysis i.e. the frequency of CD4 and CD8 T cells were confirmed by different methods used in the clinical laboratory at the site of the sample collection.

Randomization

The study subjects are part of an observational cohort without therapeutic intervention. Randomization was therefore not possible for this study.

Blinding

This study is part of an observational cohort of participants living with/without HIV with prior exposure to SARS-CoV-2. The study has no therapeutic intervention arm and therefore blinding was not possible.

Reporting for specific materials, systems and methods

We require information from authors about some types of materials, experimental systems and methods used in many studies. Here, indicate whether each material, system or method listed is relevant to your study. If you are not sure if a list item applies to your research, read the appropriate section before selecting a response.

Materials & experimental systems

n/a	Involved in the study
☐	☒ Antibodies
☐	☒ Eukaryotic cell lines
☒	☐ Palaeontology and archaeology
☒	☐ Animals and other organisms
☐	☒ Human research participants
☒	☐ Clinical data
☒	☐ Dual use research of concern

Methods

n/a	Involved in the study
☒	☐ ChIP-seq
☐	☒ Flow cytometry
☒	☐ MRI-based neuroimaging

Antibodies

Antibodies used

APC anti-human IgM Antibody BioLegend Cat # 314510 Clone # MHM-88 Lot # B269518 (Dilution: 1 in 100ul)
APC/Cyanine7 anti-human CD19 Antibody BioLegend Cat # 363010 Clone # SJ25C1 Lot #B276796 (Dilution: 1 in 100ul)
APC/Cy7 anti-human CD197 (CCR7) BioLegend Cat # 353212 Clone # G043H7 Lot #B284968 (Dilution: 1 in 50ul)
Brilliant Violet 510™ anti-human CD4 Antibody BioLegend Cat # 300546 Clone # RPA-T4 Lot #B251573 (Dilution: 1 in 200ul)
Brilliant Violet 605™ anti-human CD3 Antibody BioLegend Cat # 317322 Clone # OKT3 Lot #B310622 (Dilution: 1 in 100ul)
Brilliant Violet 650™ anti-human CD127 (IL-7Rα) Antibody BioLegend Cat # 351325 Clone # A019D5 Lot #B279597 (Dilution: 1 in 100ul)
Brilliant Violet 650™ anti-human CD3 Antibody BioLegend Cat # 317324 Clone # OKT3 Lot #B279599 (Dilution: 1 in 100ul)
Brilliant Violet 711™ anti-human CD27 Antibody BioLegend Cat # 302833 Clone # O323 Lot #B291219 (Dilution: 1 in 100ul)
Brilliant Violet 785™ anti-human CD38 Antibody BioLegend Cat # 303530 Clone # HIT2 Lot #B289087 (Dilution: 1 in 50ul)
Alexa Fluor® 700 anti-human CD45RA Antibody BioLegend Cat # 304120 Clone # HI100 Lot #B227351 (Dilution: 1 in 50ul)
PE/Cyanine7 anti-human CD45RA Antibody BioLegend Cat # 304126 Clone # HI100 Lot #B290864 (Dilution: 1 in 200ul)
Brilliant Violet 421™ anti-human CD279 (PD-1) Antibody BioLegend Cat # 329920 Clone # EH12.2H7 Lot #B285220 (Dilution: 1 in 100ul)
PE/Dazzle™ 594 anti-human CD4 Antibody BioLegend Cat # 300548 Clone # RPA-T4 Lot #B260291 (Dilution: 1 in 100ul)
PE anti-human IgD Antibody BioLegend Cat # 348204 Clone # IA6-2 Lot #B281650 (Dilution: 1 in 200ul)
APC anti-human IFN-γ Antibody BioLegend Cat # 506510 Clone # B27 Lot #B259155 (Dilution: 1 in 100ul)
Brilliant Violet 785™ anti-human CD8a Antibody BioLegend Cat # 301046 Clone # RPA-T8 Lot #B289091 (Dilution: 1 in 100ul)
Brilliant Violet 711™ anti-human CD8a Antibody BioLegend Cat # 301044 Clone # RPA-T8 Lot #B261369 (Dilution: 1 in 100ul)
Brilliant Violet 510™ anti-human CD14 Antibody BioLegend Cat # 301842 Clone # M5E2 Lot #B264335 (Dilution: 1 in 200ul)
Brilliant Violet 510™ anti-human CD19 Antibody BioLegend Cat # 302242 Clone # HIB19 Lot #B281769 (Dilution: 1 in 200ul)
Brilliant Violet 711™ anti-human CD279 (PD-1) Antibody BioLegend Cat # 329928 Clone # EH12.2H7 Lot #B286179 (Dilution: 1 in 50ul)
APC anti-human IgM Antibody BioLegend Cat # 314509 Clone # MHM-88 Lot #B269518 (Dilution: 1 in 100ul)
PE/Cyanine7 anti-human CD154 Antibody BioLegend Cat # 310832 Clone # 24-31 Lot #B188645 (Dilution: 1 in 200ul)
APC-R700 Mouse Anti-Human CD196 (CCR6) BD Biosciences Cat # 565173 Clone # 11A9 Lot #7055993 (Dilution: 1 in 25ul)
Alexa Fluor® 700 Mouse anti-Human Granzyme B BD Biosciences Cat # 560213 Clone # GB11 Lot #16941 (Dilution: 1 in 100ul)
BB515 Rat Anti-Human CXCR5 (CD185) BD Biosciences Cat # 564624 Clone # RF8B2 Lot #9212785 (Dilution: 1 in 50ul)
BV421 Mouse Anti-Human IFN-γ BD Biosciences Cat # 562988 Clone # B27 Lot #260485 (Dilution: 1 in 100ul)
PE-Cy7 Mouse Anti-Human CD25 BD Biosciences Cat # 335824 Clone # 2A3 Lot #7289693 (Dilution: 1 in 50ul)
BB700 Mouse Anti-Human CD4 BD Biosciences Cat # 566393 Clone # SK3 Lot #248679 (Dilution: 1 in 200ul)
PE-Cy™5 Mouse Anti-Human CD183 BD Biosciences Cat # 551128 Clone # 1C6/CXCR3 Lot #7040490 (Dilution: 1 in 25ul)
PE-Cy™5 Mouse Anti-Human HLA-DR BD Biosciences Cat # 562007 Clone # G46-6 Lot #45407 (Dilution: 1 in 200ul)
FITC Mouse Anti-Human TNF-α BD Biosciences Cat # 554512 Clone # MAb11 Lot #8323911 (Dilution: 1 in 400ul)
BV421 Mouse Anti-Human CD107A BD Biosciences Cat # 562623 Clone # H4A3 Lot #B207745 (Dilution: 1 in 200ul)
PerCP-eFluor 710 Anti-Human IL-2 eBioscience Cat # 46-7029-42 Clone # MQ1-17H12 Lot #E10896-1636 (Dilution: 1 in 50ul)
PerCP-eFluor 710 Anti-Human CD3 eBioscience Cat # 46-0037-42 Clone # OKT3 Lot #2073594 (Dilution: 1 in 100ul)
PE Anti-Human TIGIT eBioscience Cat # 12-9500-42 Clone # MBSA43 Lot #2093555 (Dilution: 1 in 100ul)
Monoclonal antibody specific for IFN-γ MABTECH Cat # 3420-3-250 Clone # 1-D1K Batch 97.2 (Dilution: 1 in 100ul, 1ug/ml)
Biotinylated anti-human IFN-γ MABTECH Cat # 3420-6-250 Clone # 7-B6-1 (Dilution: 1 in 1000ul, 1ug/ml)
IgG goat anti-human F(ab)'2 Jackson Immuno Cat#109-006-006 (Dilution: 1 in 1000ul)
AP-anti IgG Jackson Immuno Cat#109-055-098 (Dilution: 1 in 1000ul)

Validation

The primary antibodies used in this study were used abundantly in human subjects in published literature (https://www.thelancet.com/journals/lanhiv/article/PIIS2352-3018(20)30069-2/fulltext) and were validated by manufacturers for that application; this information is provided on the manufacturers' website and product information datasheets as follow:

APC anti-human IgM Antibody BioLegend Cat # 314510
https://www.biolegend.com/en-us/search-results/apc-anti-human-igm-antibody-2881?GroupID=BLG4120

APC/Cyanine7 anti-human CD19 Antibody BioLegend Cat # 363010
https://www.biolegend.com/en-us/products/apc-cyanine7-anti-human-cd19-antibody-10302

APC/Cy7 anti-human CD197 (CCR7) BioLegend Cat # 353212
https://www.biolegend.com/en-us/products/apc-cyanine7-anti-human-cd197-ccr7-antibody-7524

Brilliant Violet 510™ anti-human CD4 Antibody BioLegend Cat # 300546
https://www.biolegend.com/en-us/products/brilliant-violet-510-anti-human-cd4-antibody-9598

Brilliant Violet 605™ anti-human CD3 Antibody BioLegend Cat # 317322
https://www.biolegend.com/en-us/products/brilliant-violet-605-anti-human-cd3-antibody-7666

Brilliant Violet 650™ anti-human CD127 (IL-7Rα) Antibody BioLegend Cat # 351325
https://www.biolegend.com/en-us/products/brilliant-violet-650-anti-human-cd127-il-7ralpha-antibody-7673

Brilliant Violet 650™ anti-human CD3 Antibody BioLegend Cat # 317324
https://www.biolegend.com/en-us/products/brilliant-violet-650-anti-human-cd3-antibody-7667

Brilliant Violet 711™ anti-human CD27 Antibody BioLegend Cat # 302833
https://www.biolegend.com/en-us/products/brilliant-violet-711-anti-human-cd27-antibody-7935

Brilliant Violet 785™ anti-human CD38 Antibody BioLegend Cat # 303530
https://www.biolegend.com/en-us/products/brilliant-violet-785-anti-human-cd38-antibody-7971

Alexa Fluor® 700 anti-human CD45RA Antibody BioLegend Cat # 304120
https://www.biolegend.com/en-us/products/alexa-fluor-700-anti-human-cd45ra-antibody-3421

PE/Cyanine7 anti-human CD45RA Antibody BioLegend Cat # 304126
https://www.biolegend.com/en-us/products/pe-cyanine7-anti-human-cd45ra-antibody-7055

Brilliant Violet 421™ anti-human CD279 (PD-1) Antibody BioLegend Cat # 329920
https://www.biolegend.com/en-us/products/brilliant-violet-421-anti-human-cd279-pd-1-antibody-7191

PE/Dazzle™ 594 anti-human CD4 Antibody BioLegend Cat # 300548
https://www.biolegend.com/en-us/products/pe-dazzle-594-anti-human-cd4-antibody-9780

PE anti-human IgD Antibody BioLegend Cat # 348204 Clone # IA6-2
https://www.biolegend.com/en-us/products/pe-anti-human-igd-antibody-6532

APC anti-human IFN-γ Antibody BioLegend Cat # 506510
https://www.biolegend.com/en-us/products/apc-anti-human-ifn-gamma-antibody-1533

Brilliant Violet 785™ anti-human CD8a Antibody BioLegend Cat # 301046 https://www.biolegend.com/en-ie/products/brilliant-violet-785-anti-human-cd8a-antibody-7919

Brilliant Violet 711™ anti-human CD8a Antibody BioLegend Cat # 301044
https://www.biolegend.com/en-ie/products/brilliant-violet-711-anti-human-cd8a-antibody-7929

Brilliant Violet 510™ anti-human CD14 Antibody BioLegend Cat # 301842 https://www.biolegend.com/en-ie/products/brilliant-violet-510-anti-human-cd14-antibody-8001

Brilliant Violet 510™ anti-human CD19 Antibody BioLegend Cat # 302242 https://www.biolegend.com/en-ie/products/brilliant-violet-510-anti-human-cd19-antibody-8004

Brilliant Violet 711™ anti-human CD279 (PD-1) Antibody BioLegend Cat # 329928 https://www.biolegend.com/en-ie/products/brilliant-violet-711-anti-human-cd279-pd-1-antibody-7945

APC anti-human IgM Antibody BioLegend Cat # 314509
https://www.biolegend.com/en-ie/products/apc-anti-human-igm-antibody-2881

PE/Cyanine7 anti-human CD154 Antibody BioLegend Cat # 310832 https://www.biolegend.com/en-us/search-results/pe-cyanine7-anti-human-cd154-antibody-9233?GroupID=BLG2341

APC-R700 Mouse Anti-Human CD196 (CCR6) BD Biosciences Cat # 565173 https://www.bdbiosciences.com/en-eu/products/reagents/flow-cytometry-reagents/research-reagents/single-color-antibodies-ruo/apc-r700-mouse-anti-human-cd196-ccr6.565173

Alexa Fluor® 700 Mouse anti-Human Granzyme B BD Biosciences Cat # 560213 https://www.bdbiosciences.com/en-eu/products/reagents/flow-cytometry-reagents/research-reagents/single-color-antibodies-ruo/alexa-fluor-700-mouse-anti-human-granzyme-b.560213

BB515 Rat Anti-Human CXCR5 (CD185) BD Biosciences Cat # 564624

https://www.bdbiosciences.com/en-us/products/reagents/flow-cytometry-reagents/research-reagents/single-color-antibodies-ruo/bb515-rat-anti-human-cxcr5-cd185.564625

BV421 Mouse Anti-Human IFN-γ BD Biosciences Cat # 562988
https://www.bdbiosciences.com/en-us/products/reagents/flow-cytometry-reagents/research-reagents/single-color-antibodies-ruo/bv421-mouse-anti-human-ifn.562988

PE-Cy7 Mouse Anti-Human CD25 BD Biosciences Cat # 335824
https://www.bdbiosciences.com/eu/reagents/clinical/reagents/single-antibodies/cd25-pe-cytrade7-2a3/p/335824

BB700 Mouse Anti-Human CD4 BD Biosciences Cat #
https://www.bdbiosciences.com/en-us/products/reagents/flow-cytometry-reagents/research-reagents/single-color-antibodies-ruo/bb700-mouse-anti-human-cd4.566393

PE-Cy™5 Mouse Anti-Human CD183 BD Biosciences Cat # 551128
https://www.bdbiosciences.com/en-eu/products/reagents/flow-cytometry-reagents/research-reagents/single-color-antibodies-ruo/pe-cy-5-mouse-anti-human-cd183.551128

PE-Cy™5 Mouse Anti-Human HLA-DR BD Biosciences Cat # 562007
https://www.bdbiosciences.com/en-eu/products/reagents/flow-cytometry-reagents/research-reagents/single-color-antibodies-ruo/pe-cy-5-mouse-anti-human-hla-dr.562007

FITC Mouse Anti-Human TNF-α BD Biosciences Cat # 554512
https://www.bdbiosciences.com/en-eu/products/reagents/flow-cytometry-reagents/research-reagents/single-color-antibodies-ruo/fitc-mouse-anti-human-tnf.554512

PerCP-eFluor 710 Anti-Human IL-2 eBioscience Cat # 46-7029-42
https://www.thermofisher.com/antibody/product/IL-2-Antibody-clone-MQ1-17H12-Monoclonal/46-7029-42

PerCP-eFluor 710 Anti-Human CD3 eBioscience Cat # 46-0037-42
https://www.thermofisher.com/antibody/product/CD3-Antibody-clone-OKT3-Monoclonal/46-0037-42

PE Anti-Human TIGIT eBioscience Cat # 12-9500-42
https://www.thermofisher.com/antibody/product/TIGIT-Antibody-clone-MBSA43-Monoclonal/12-9500-42

BV421 Mouse Anti-Human CD107A BD Biosciences Cat # 562623
https://www.bdbiosciences.com/en-us/products/reagents/flow-cytometry-reagents/research-reagents/single-color-antibodies-ruo/bv421-mouse-anti-human-cd107a.566261

Monoclonal antibody specific for IFN-γ MABTECH Cat # 3420-3-250
https://www.mabtech.com/products/anti-human-ifn-gamma-antibody-1-d1k-purified-3420-3

Biotinylated anti-human IFN-γ MABTECH Cat # 3420-6-250
https://www.mabtech.com/products/anti-human-ifn-gamma-antibody-7-b6-1-biotinylated-3420-6

IgG goat anti-human F(ab)'2 Jackson Immuno Cat#109-006-006
https://www.jacksonimmuno.com/catalog/products/109-006-006

AP-anti IgG Jackson Immuno Cat#109-055-098
https://www.jacksonimmuno.com/catalog/products/109-055-098

All antibodies described here have been further optimized for an appropriate concentration by testing several dilutions.

Eukaryotic cell lines

Policy information about cell lines

Cell line source(s)	HEK293T/17 cells (ATCC CRL-11268), HeLa-ACE-2 cells (James Voss Laboratory, The Scripps Research Institute, described in: https://science.sciencemag.org/content/369/6506/956)
Authentication	None of the cell lines were authenticated.
Mycoplasma contamination	Both cell lines were tested mycoplasma-free by PCR
Commonly misidentified lines (See ICLAC register)	None

Human research participants

Policy information about studies involving human research participants

Population characteristics	A total of n=47 HIV positive and n=35 HIV negative subjects with recovered confirmed and/or suspected COVID-19 disease were included. Sixteen demographically age-, sex- and lifestyle-matched HIV-1 seropositive individuals were included for comparison, from whom sample were collected between (February 2017-November 2019; pre-pandemic). Supplementary Table 1 includes the cohort demographics and clinical characteristics. Comparison analyses according to gender or age were included in the figures and noted in the results section. The case definition for COVID-19 was described as of 03 December 2020, European Centre for Disease Prevention and Control [https://www.ecdc.europa.eu/en/covid-19/surveillance/casedefinition]. Severity of COVID-19 was classified according to the WHO (World Health Organisation) clinical progression scale (Aitken et al ., 2020).
Recruitment	HIV seronegative adults (>18 years of age, comprising hospital-based healthcare workers) and chronically HIV infected patients (on antiretroviral treatment for at least 2 years with undetectable HIV RNA) with prior confirmed or suspected COVID-19 disease were recruited at the Mortimer Market Centre for Sexual Health and HIV Research and the Royal Free Hospital (London, UK) following written informed consent as part of a study approved by the local ethics board committee. Confirmed SARS-CoV-2 infection by SARS-CoV-2 PCR and/or Roche antibody tests was declared by the participants, who were asked to provide details on the timing and nature of symptoms. The case definition for COVID-19 was described as of 03 December 2020, European Centre for Disease Prevention and Control [https://www.ecdc.europa.eu/en/covid-19/surveillance/casedefinition]. Eligible HIV-positive participants were identified by clinicians providing HIV care, indicating that they are willing to be contacted by the research team, and HIV-negative controls (health care workers) through advertising in the local clinics. Self-selection bias may have occurred due to the nature of recruitment.<br><br>Potential bias that could impact results include biological factors including age and gender of the HIV-positive participants. We have provided detailed analyses of the SARS-CoV-2 antibody and T cell responses according gender and age (Supplementary Figure 1-3, and Supplementary Figure 6)
Ethics oversight	The samples were collected using the protocols for the human study which were approved by the local Research Ethics Committee (REC) – Berkshire (REC 16/SC/0265). The study conformed to the Helsinki declaration principles and Good Clinical Practice (GCP) guidelines and all subjects enrolled into the study provided written informed consent.

Note that full information on the approval of the study protocol must also be provided in the manuscript.

Flow Cytometry

Plots

Confirm that:

- [x] The axis labels state the marker and fluorochrome used (e.g. CD4-FITC).
- [x] The axis scales are clearly visible. Include numbers along axes only for bottom left plot of group (a 'group' is an analysis of identical markers).
- [x] All plots are contour plots with outliers or pseudocolor plots.
- [x] A numerical value for number of cells or percentage (with statistics) is provided.

Methodology

Sample preparation	For Phenotypic flow cytometric analysis : Cryopreserved PBMCs were thawed and rested for one hour at 37 °C in complete RPMI medium (RPMI supplemented with Penicillin-Streptomycin, L-Glutamine, HEPES, non-essential amino acids, 2-Mercaptoethanol, and 10% Fetal bovine serum (FBS)). Cells were then washed, resuspended in PBS, and surface stained at 4° C for 20 min with different combinations of antibodies in the presence of fixable live/dead stain (Invitrogen). Cells were then fixed and permeabilized for detection of intracellular antigens. The Foxp3 intranuclear staining buffer kit (eBioscience) was used according to the manufacturer's instructions for the detection of intranuclear markers.<br><br>For ICS assay: Cryopreserved PBMCs were thawed and rested overnight at 37 °C and 5% carbon dioxide in complete RPMI medium. After overnight rest, PBMCs were stimulated for 6 h with 2µg/mL of SARS-CoV-2 peptide pools, Influenza, HIV-1 Gag or cytomegalovirus (CMV)-pp65 peptide pools, or with 0.005% dimethyl sulphoxide (DMSO) as a negative control in the presence of αCD28/αCD49d co-Stim antibodies (1 µg ml–1) GolgiStop (containing Monensin, 2 µmol/L), GolgiPlug (containing brefeldin A, 10 µg ml–1) (BD Biosciences) and anti-CD107α BV421 antibody (BD Biosciences). After stimulation, cells were washed and stained with anti-CCR7 (BioLegend) for 30 min at 37 °C and then surface stained at 4°C for 20 min with different combinations of surface antibodies in the presence of fixable live/dead stain (Invitrogen). Cells were then fixed and permeabilised (CytoFix/CytoPerm; BD Biosciences) followed by intracellular cytokine with IFN-g APC, CD154 PE-Cy7 (BioLegend), TNF-α FITC (BD Biosciences) and PerCP-eFluor 710 IL-2 (eBioscience).
Instrument	BD Fortessa X20 using BD FACSDiva8.0 (BD Bioscience)
Software	FlowJo 10 (TreeStar) and Cytobank platform (https://cytobank.org)
Cell population abundance	Sorting was not involved in the study.
Gating strategy	For all the flow cytometric analysis, cells were first gated on single lymphocyte population by side and forward side scatter,

followed by live cells gating by excluding dead, CD19 and CD14 cells. Following live CD3+ cells gating, cells were gated on CD4 T cells (CD4+CD8-) or CD8 T cells (CD4-CD8+). The cytokine positive/negative cells were gated according to corresponding negative controls, known as control samples. The gating strategy for detection of virus specific responses are described in the figure legends and method section. Flow plot representative examples are included in the main figures and Supplementary Fig.7a-d.

☒ Tick this box to confirm that a figure exemplifying the gating strategy is provided in the Supplementary Information.
